# Paper-Based Working Electrodes Coated with Mercury or Bismuth Films for Heavy Metals Determination

**DOI:** 10.3390/bios10050052

**Published:** 2020-05-13

**Authors:** Alberto Sánchez-Calvo, Maria Carmen Blanco-López, Agustín Costa-García

**Affiliations:** Departamento de Química Física y Analítica, Facultad de Química, Universidad de Oviedo, 33006 Oviedo, Spain; albertosc14@hotmail.com (A.S.-C.); costa@uniovi.es (A.C.-G.)

**Keywords:** paper electrodes, bismuth films, mercury films, low-cost analysis, heavy metal determination

## Abstract

Paper-based carbon working electrodes were modified with mercury or bismuth films for the determination of trace metals in aqueous solutions. Both modification procedures were optimized in terms of selectivity and sensitivity for the determination of different heavy metals, aiming their simultaneous determination. Cd (II), Pb (II) and In (III) could be quantified with both films. However, Cu (II) could not be determined with bismuth films. The modification with mercury films led to the most sensitive method, with linear ranges between 0.1 and 10 µg/mL and limits of detection of 0.4, 0.1, 0.04 and 0.2 µg/mL for Cd (II), Pb (II), In (III) and Cu (II), respectively. Nevertheless, the bismuth film was a more sustainable alternative to mercury. Tap-water samples were analyzed for the determination of metals by standard addition methodology with good accuracy, by using a low-cost and easily disposable paper-based electrochemical platform. This system demonstrated its usefulness for monitoring heavy metals in water.

## 1. Introduction

Electrochemistry research is looking at new materials and surfaces for sustainable technological applications in microelectronics, energy or in the development of sensors. In this field, surface characteristics of the electrodes are important for electron transfer steps or non-faradaic interactions, like adsorption or ion-pair formation [1,2]. Mercury electrodes have been widely used for several decades because of their large cathodic window, reproducibility and low background [3,4]. However, mercury is a dangerous heavy metal because of its toxicity and bio-accumulation in many species. This has triggered the search for less-toxic alternatives [5,6,7]. Nowadays, carbon is one of the most advantageous materials for low-cost and flexible-design electrodes. Glassy carbon, carbon paste or screen-printed carbon electrodes (SPCE) are easy to fabricate and to modify for sensor development [8,9,10]. With the use of carbon electrodes, advantageous properties of mercury surfaces, such as interactions with thiol peptides or potential windows down −2.5 V (vs. Ag/AgCl), are restricted [2,11,12,13,14]. However, mercury can be electrochemically deposited on the conductive carbon surface, forming a thin film which can have the same applications as conventional mercury electrodes [15,16,17], but significantly reducing the required amount. Determination of trace metals by stripping voltammetry is one of the most developed applications on this surface because of the high affinity of mercury for metals [11,17,18,19]. The procedure followed for determining heavy metals with stripping techniques involves the preconcentration of metal species on a solid electrode surface, followed by a selective oxidation of each metal during an anodic potential sweep. The peak current, being proportional to the concentration of the heavy metal in the solution, is recorded.

Nowadays, bismuth films are being used as an alternative to mercury films, with the objective of using a more environmentally friendly platform [20,21,22,23,24,25]. Bismuth has very low toxicity and possesses suitable electrochemical properties, such as a wide range of operating negative potentials, low background currents and the ability to electrodeposit elements on its surface by formation of intermetallic compounds or fused alloys. It has also a high-quality stripping performance, and therefore is good for the replacement of mercury at the determination of heavy metals by stripping voltammetry [23,26]. Likewise, bismuth films can also be formed by different ways on carbon substrates, mainly by applying a negative potential in situ, (i.e., bismuth is in the sample solution together with the analytes) [27,28,29,30,31,32], or ex situ by previous deposition, in a solution different from this of the analyte [33,34,35,36]. Recently, heavy metals determination by using bismuth nanoparticles was documented [37,38].

Bulk bismuth is the semimetal with the smallest electron effective mass [39]. This confers advantages like more pronounced quantum confinement effects becoming a good option in the form of a nanostructure. There are several chemical ways to form nanoparticles [40], like pyrolysis of bismuth [41], adding reducing agents or photochemical activation [42]. Reductive methods like adding sodium borohydride to bulk bismuth allow to control nanoparticles in terms of size and shape [43]. However, bismuth films can be generated electrochemically, with the advantage of faster in situ modification, employing less reagents.

Bismuth and mercury films have been successfully formed on glassy carbon, carbon paste and screen-printed carbon electrodes (SPCEs) [11,31,44,45,46,47,48,49], for the determination of heavy metals such as Cd (II), Pb (II) or Cu (II), and they were also successfully applied to the determination of Tl (I) [50]. Recent trends in analytical chemistry encourage the development of simpler alternatives that could also be used in developing countries. Conventional glassy carbon and carbon paste electrodes are not very appropriate for decentralized analysis, and although screen-printed electrodes are an interesting alternative, very often they are developed on ceramic substrates. Searching for low-cost and easy-to-dispose materials is then of enormous interest.

The use of paper as substrate for sensors is considered a good option for electrochemical measurements [51,52,53,54,55]. It is composed of cellulose fibers which confer properties like roughness and hydrophilicity, becoming easy to handle, with the possibility to form three-dimensional sensors [40,41,42,43]. Paper can also be modified by the addition of (i) hydrophobic materials, like wax, to form barriers that allow customization of the electrochemical cell, (ii) appropriate inks to have a conductive surface or (iii) nanostructures to improve selectivity and/or sensitivity [56,57,58,59,60]. Research literature on the determination of heavy metals by using paper electrodes is scarce. Reports include colorimetric or electrochemical determinations [58,61,62]. Films (e.g., bismuth) generated on paper can also be modified with nanomaterials, to improve the sensitivity of the methodology [63].

In this work, we studied the ex situ deposition of bismuth films on a working paper-based electrode developed in previous works [59,64,65] and compared the performance with that obtained with more traditional mercury films.

These electrodes were tested with Cd (II), Pb (II), In (III) and Cu (II) aqueous solutions. The working paper electrode prepared was placed over the working electrode of a screen-printed carbon card, having also an auxiliary and reference electrode which can be reutilized without any interference between measurements. On this platform, the bismuth and mercury films were electrodeposited from the corresponding salts by applying a negative potential. After that, heavy metals previously preconcentrated were analyzed by anodic stripping voltammetry. With this method, the paper electrode used can be removed, allowing its use for other analysis without any contamination from the last measurement. Bismuth films were chosen as an environmentally friendly alternative to mercury. Both films were compared in terms of sensitivity and selectivity. The use of these kind of films allows the development of sensors for heavy metals, by using paper-based carbon electrodes and low volumes of reagents for the formation of films or the sample analysis. This results on great economical savings and lower toxic waste generated. The electrochemical detection allows the characterization of heavy metals by using only the previously formed film, instead of using different reagents for every heavy metal, as most colorimetric sensors do [66]. This also lowers the economic cost. Besides, the small size of these foldable paper electrodes makes them portable and suitable for easy waste treatment. Paper acts as storage, concentrating heavy metals in a porous solid substrate.

## 2. Materials and Methods

### 2.1. Reagents and Materials

Hg (II) acetate, sodium sulfate and Cu (II) nitrate trihydrate were purchased from Sigma-Aldrich (USA). Cd (II) standard solution (1000 µg/mL) was acquired from Merck KGaA (Germany) (https://www.merckgroup.com/en). Standard solutions of lead and bismuth for ICP were acquired from Fluka Analytical (https://www.lab-honeywell.com/products/brands/fluka/). In (III) chloride was acquired from Alfa Aesar (Germany) (https://www.alfa.com/es/). Ultrapure water (18.2 MΩ) was directly taken from a Millipore Direct-Q^®^ 3 UV purification system from Millipore Ibérica (Spain) (https://www.tecnoaqua.es/empresas/millipore-iberica-sa). N,N-dimethylformamide anhydrous (DMF) was acquired from Sigma-Aldrich (https://www.sigmaaldrich.com).

A 0.1 M acetate buffer solution was prepared by mixing 0.1 M acetic acid with 0.5 M sodium sulphate as background electrolyte and adjusting the pH with sodium hydroxide, until pH 4.0. A 10^−3^ M bismuth solution was prepared in the acetate buffer solution, whereas a 10^−3^ M solution of mercury was prepared by solving the corresponding amount of mercury (II) acetate in 0.1 M HCl. All the solutions of heavy metals were prepared in 0.1 M acetate buffer pH 4, 0.5 M in sodium sulphate (employed as background electrolyte).

Carbon paste (ref. C10903P14) was acquired from Gwent group (United Kingdom) (http://www.gwent.org/). A wax printer (Xerox Colorqube 8570) was used to wax-print the paper. A thermostat model (Nabertherm d-2804) was used to melt the wax. An ultrasonic bath sonicator (Elmasonic P) was employed to homogenize carbon ink solutions. The spray adhesive 3M Spray Mount^TM^ was acquired from a local store.

Screen-printed electrode cards were obtained from Metrohm.Dropsens (http://www.dropsens.com/) (SPCEs, ref. DRP-110, Spain). Working and auxiliary electrodes were made of carbon ink, and the pseudo-reference electrode was silver. They were connected to the potentiostat by a DSC connector (ref. DRP-DSC) from the same company. Electrochemical measurements were carried out with a potentiostat (Autolab, PGSTAT 10) controlled by the Autolab GPES software.

### 2.2. Fabrication of Paper-Based Electrodes

Paper-based working electrodes were prepared by a procedure previously developed in our group [64]. Hydrophobic wax patterns designed by Inkscape software were printed on chromatography paper Whatman Grade 1 as cellulose substrate. A temperature of 80 °C was applied on the paper until wax was melted, followed by cooling at room temperature.

The next step was the modification of the paper by the addition of 2 µL of a carbon ink suspension by drop casting on one of the sides (“bottom side”). The measuring solutions were added by the other side (“upper side”). The carbon ink suspensions used to modify the chromatography paper were made by dilution of commercial carbon paste in anhydrous N,N-dimethylformamide (DMF) to a final concentration of 40% (w/w). Homogenization was performed by sonication for 1 h.

**Figure 1:**
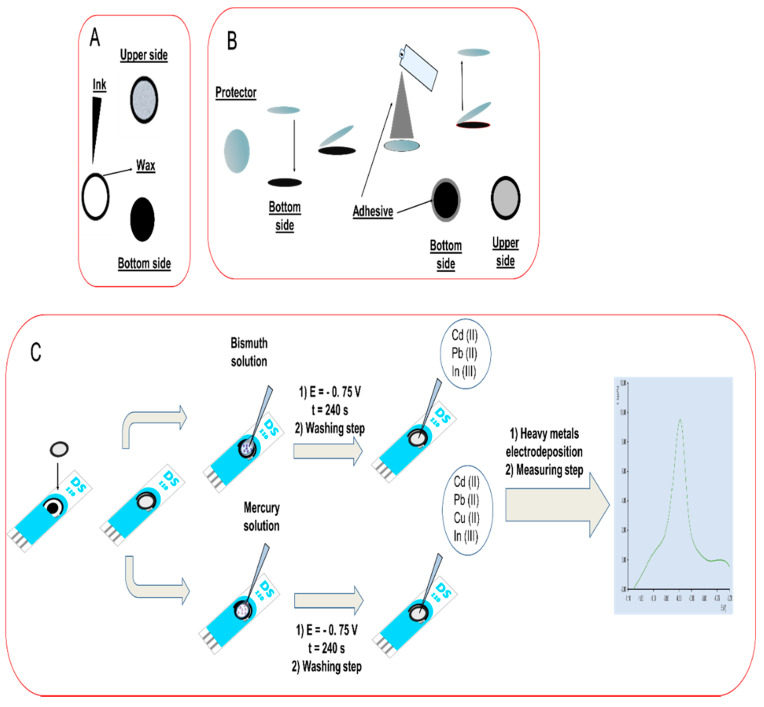
Schematic diagram showing the preparation of the paper-based electrodes. (**A**) Modification of cellulose substrate by addition of carbon ink on one side, having the platform with two visually different sides. Solution was added with a micropipette through the upper side. (**B**) Adhesive spraying on the protected working electrode. (**C**) Overlapping process of the bottom side of the paper electrode (the one with the ink) on the screen-printed card and modification with mercury (II) acetate or bismuth (III) standard solutions.

The whole process of preparation of the paper platform is shown in Figure 1. Each cellulosic electrode was cut to obtain a circle with a diameter of 4 mm. The area was wide enough to cover the working electrode (WE) of a screen-printed platform, as well as the ceramic surface between the WE and the auxiliary (AE) and reference (RE) electrodes. The area of the cellulosic working electrode was covered with a protective polymeric circular film before spraying with an adhesive. Then, after removing the protective film, the working paper-based electrode was placed over the WE of a screen-printed carbon electrode card (SPCE). Following this procedure, the carbon ink in the paper and that of the WE of the SPCE were in contact. Moreover, there was no interference from the adhesive spray, which was only spread on the crown surrounding the protector. Thus, the WE of the SPCE acted just as a connection. Before performing the measurements, it was verified that there was full overlap, and also that the solution added on the surface of the paper WE did not wet the WE of the SPCE. By ensuring this, the SPCE could be reused, without any interference from previous measurements.

### 2.3. Modification of Paper Electrodes: Deposition of Mercury and Bismuth Films

Mercury and bismuth films were generated in the paper electrode by using standard solutions of 10^−3^ M Bi (III) in 0.1 M acetate buffer (with 0.5 M sodium sulphate as background electrolyte) pH 4, or 10^−3^ M Hg (II) in 0.1 M HCl. A potential of - 0.75 V was applied in both cases. Then, the paper electrode was washed with Milli-Q water and was ready for the measurements.

### 2.4. Electrochemical Procedures

Linear sweep voltammetry was used to characterize the redox processes of each heavy metal (Cu (II), In (III), Pb (II) and Cd (II)) and to optimize the formation of the film on the cellulose-based electrode. A potential of −1.2 V was applied to preconcentrate the analytes before the measurement. Then, the reduced analyte was stripped away according to the next parameters: initial potential (E_i_) = -1.2 V, final potential (E_f_) = 0 V, step potential (E_s_) = 2 mV and scan rate (*v*) = 50 mV/s.

All heavy metals’ measurements were carried out in 0.1 M acetate buffer pH 4, 0.5 M in sodium sulphate, employed as background electrolyte.

Relative standard deviations (RSDs) were calculated for all analytes at concentrations located in the middle of the linear range of each analyte. Different paper electrodes were tested (*n* = 3), using different SPCEs for different analytes.

### 2.5. Water Analysis

Samples of tap water were collected in our laboratory for heavy metal determination. They were spiked with standards of Cd (II) and Pb (II) before their analysis. The solutions were analyzed by anodic stripping linear sweep voltammetry, but the parameters for the modification and measurement steps in water samples were different depending on the type of film used.

For the determination at paper-based electrodes with mercury films, water samples were spiked with different volumes of 10 µg/mL of Cd (II) and Pb (II). The spiked samples were diluted 1:10 in 0.1 M acetate buffer, pH 4, with 0.5 M sodium sulphate used as background electrolyte. Metals were preconcentrated on paper electrodes modified with mercury films, by applying a potential of -1.2 V for 600 s. Then, deposited heavy metals were stripped away by linear sweep voltammetry by applying the following conditions: E_i_ = −1.2 V, E_f_ = 0 V, E_s_ = 2 mV and *v* = 50 mV/s.

On the other hand, samples of tap water collected to be quantified by using paper-based electrodes modified with bismuth films were spiked with different volumes of a standard solution of 25 µg/mL of Zn (II), Cd (II) and Pb (II). The spiked tap-water sample was 1:10 diluted in 0.1 M acetate buffer, pH 4, with 0.5 M sodium sulphate. A preconcentration potential of -1.3 V was applied for 600 s according to a procedure previously published [36]. Afterward, deposited metals were stripped anodically by sweeping the potential linearly from −1.3 to −0.6 V at 50 mV/s, with 2 mV of step potential. Measurements were recorded in triplicate.

## 3. Results and Discussions

### 3.1. Electrochemical Characterization of Mercury/Bismuth Films at Paper Electrodes

The mercury or bismuth films electrochemically formed on the carbon ink deposited at the cellulose matrix of paper electrodes were characterized by linear sweep voltammetry (LSV). The optimization of the film formation method was done by measuring the intensity of the oxidation current of a Cd (II) solution deposited on the paper electrode after the film formation. Cd (II) solution was chosen because, among the heavy metals tested, this was the one with the most negative reduction potential. A negative potential of −1.2 V was used to preconcentrate this metal for different deposition times, varying from 60 to 300 s. Then, the reduced Cd (0) was stripped away by scanning the potential anodically. Regarding the film generation, the application of −0.75 V for 240 s on an aliquot of 40 µL of 10^−3^ M of bismuth or mercury standard solutions was the option that produced the most precise signals with highest intensities for both mercury and bismuth films. Higher deposition times did not increase the intensity of the oxidation currents. Therefore, this time was used for the film deposition at the analysis of the heavy metals in water samples.

### 3.2. Determination of Heavy Metals at Paper-Based Electrodes with Mercury/Bismuth Films

Standard solutions of heavy metals such as Cd (II), Pb (II), In (III) and Cu (II) were prepared in 0.1 M acetate buffer pH 4 with 0.5 M sodium sulphate. This salt was used to increase the conductivity of the solution. Firstly, all of them were electrochemically characterized by linear sweep voltammetry on an individual base. Standard solutions of each analyte were added on paper electrodes modified with mercury films, to perform individual measurements. A potential of −1.2 V was applied for the preconcentration step, and then the analyte was stripped away by applying an anodic potential scan from −1.2 to 0 V. Different preconcentration times were tested, varying from 240 to 720 s, with 600 s being the option with the highest peak current intensity for Cd (II), In (III), Pb (II) and Cu (II). Their oxidation peak potentials were −905, −800, −720 and −180 mV, respectively, on paper electrodes modified with mercury films. 

In order to evaluate a more sustainable alternative with less toxicity, the same procedure was carried out on paper electrodes modified with bismuth films. Following a similar procedure, in this case, the oxidation peaks for Cd (II), In (III) and Pb (II) occurred at −1000, −920 and −780 mV. Cu (II) could not be detected on paper-based electrodes modified with bismuth films because its analytical signal overlapped with a bismuth oxidation peak that appeared at −300 mV. Cd (II), In (III) and Pb (II) were successfully detected in both films. For a 10 µg/mL concentration of Pb (II), oxidation intensity currents of 8 and 19 µA were obtained in paper electrodes modified with bismuth and mercury films, respectively. For In (III), this was also higher in paper electrodes modified with mercury films, obtaining values of 10 µA for a concentration of 2.5 µg/mL, and 2.5 µA for 2 µg/mL in bismuth films. However, in the case of Cd (II), the intensity of peak currents was nearly similar in both cases (19 and 16 µA, respectively, for a concentration of 10 µg/mL). However, the linear range is wider in paper electrodes modified with mercury films, and the limits of detection are lower.

Calibrations curves for the single determination of heavy metals (Cd (II), In (III), Pb (II) and Cu (II)) were carried out by anodic stripping linear sweep voltammetry by employing the same parameters (*n* = 3). Apart from LSV, different electrochemical techniques (differential pulse voltammetry (DPV) and square wave voltammetry (SWV)) were tested. LSV was the best option because of the good reproducibility obtained with all analytes tested. Thus, LSV was employed for the remaining work. Table 1 shows the equations for the calibration curves, along with analytical characteristics, such as the linear ranges and limits of detection obtained with paper electrodes modified with mercury or bismuth films. Similar slopes for Cd (II) and Pb (II) obtained in mercury films can be explained because they have similar diffusion coefficients in mercury (1.5 × 10^−5^ cm^2^/s for Cd (II), 1.25 × 10^−5^ for Pb (II)), respectively [67].

Therefore, mercury films showed higher slopes for all the analytes, except for Cd (II). However, Cd (II) in bismuth films is measured in a narrower linear range. In conclusion, paper electrodes with mercury films have higher sensitivity for all analytes tested.

The limits of detection (LOD) for Cd (II), Pb (II), In (III) and Cu (II) were calculated as the concentration corresponding to a signal equivalent to *3S_a_/m,* where *S_a_* is the standard deviation of the blank (intercept), and *m* is the slope of the calibration line of paper electrodes with mercury or bismuth films. The lowest LODs were achieved in all cases for the paper electrodes modified with mercury films. The relative standard deviation (RSD) corresponding to a concentration located in the middle of the linear range for each analyte is also reported. They were done by using the same screen-printed electrode card for all the paper electrodes. Nevertheless, the RSD did not change by using different SPCEs. Care should be taken in fixing and removing the paper electrodes, in order to avoid ink losses and therefore, connection damage. In this way, screen-printed cards can be reused, at least for ca. seven measurements.

Although for Cd (II) the methodology that employs paper electrodes modified with mercury films has a better limit of detection (LOD), the precision was better for mercury films. In the case of Pb (II), both sensitivity and reproducibility were better when mercury films were used. In (III) had the best LOD for both types of films, being lower for mercury films. The RSDs are acceptable. Cu (II) was only measured on paper electrodes modified with mercury films, obtaining the a wide linear range. Therefore, in general, mercury films gave better results with higher sensitivity and allowed the determination of Cu (II). However, bismuth films showed reliable results with the advantage of being a material less toxic than mercury.

Among all the heavy metals tested, Cd (II) and Pb (II) are the most toxic, having legal limits more restrictive in drinking water in comparison with In (III) and Cu (II). Legislation sets limits of 5 and 10 µg/L for Cd (II) and Pb (II), respectively, whilst Cu (II) has a limit of 2000 µg/L, making Cd (II) and Pb (II) the most important ones for quality analysis [68,69]. There is no established limit for In (III), an element whose toxicity and environmental effects are still not confirmed, but its use as a component in alloys and also in the semiconductor industry makes its determination useful for quality control purposes. With this procedure, Cd (II) and Pb (II) provided reliable results (using mercury and bismuth films) in terms of linear range, reproducibility or sensitivity, concluding that they can be determined in water samples different from drinking water because of the legislation limits. However, our research group has proved that it is possible to decrease 800-fold the LOD of the determination of a contaminant (diclofenac) in waters with this type of electrodes, by simply depositing several aliquots of the sample and allowing the preconcentration in the paper matrix before launching the measurement [70]. This opens the possibility to improve the sensitivity of the detections if required. In the case of In (III), the methodology also showed reliable results, resulting in an In (III) sensor in aqueous solutions for the semiconductor industry. Finally, in the case of Cu (II), mercury-film paper-based electrodes can be used as a low-cost sensor for drinking water.

**Figure 2:**
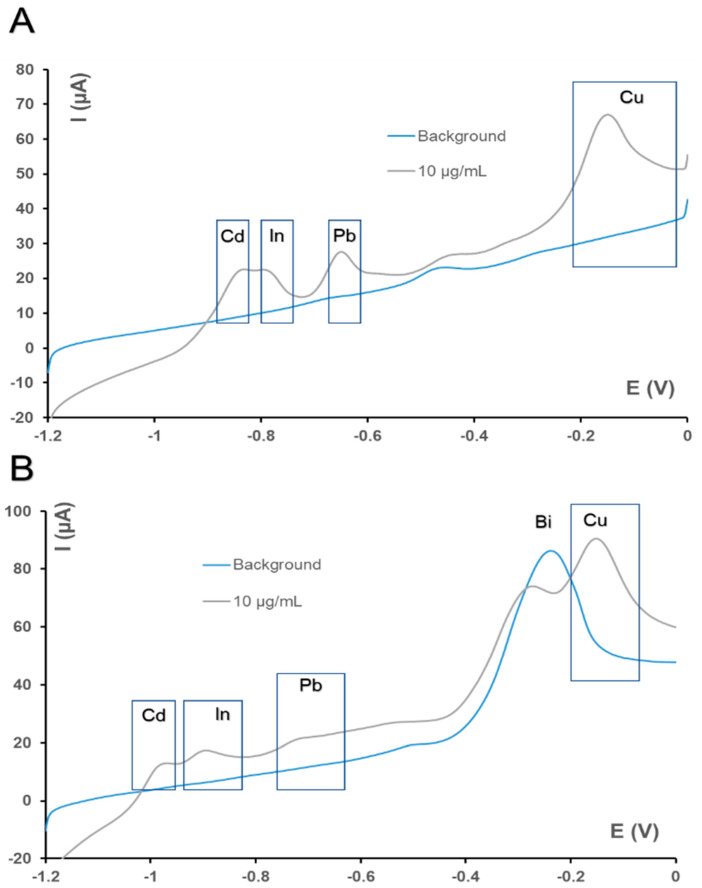
Linear sweep voltammograms for Pb (II), Cd (II), Cu (II) and In (III) (10 µg/mL) solutions recorded in paper-based electrodes modified with (**A**) mercury or (**B**) bismuth films. Preconcentration step: E_d_ = −1.2 V, t_d_ = 600 s). Stripping step: E_i_ = −1.2 V, E_f_ = 0 V, E_s_ = 2 mV, *v* = 50 mV/s).

On the other hand, in order to study the possibility of making multianalyte determination, with simultaneous measurements for all heavy metals studied, solutions containing different concentrations of Cd (II), In (III), Pb (II) and Cu (II) were prepared. Determinations were carried out by anodic stripping linear sweep voltammetry, employing the same parameters as before. It could be observed that the oxidation reactions for Cd and In for concentrations of 10 µg/mL in paper electrodes with mercury films overlapped, with potentials of −845 and −780 mV, respectively. In the case of bismuth films, another overlap between the anodic peaks of Bi and Cu (with −234 and −140 mV respective peak potentials) was observed. However, Pb (II) and Cd (II) could be identified with enough resolution. Linear sweep voltammograms recorded in solutions containing Cd (II), In (III), Pb (II) and Cu (II) for both films are shown in Figure 2.

### 3.3. Analysis of Water: Determination of Cd (II) and Pb (II)

Water samples obtained from our laboratory were collected as a substitute for sewage-water samples and spiked for Cd (II) and Pb (II) determination, using paper electrodes modified with mercury and bismuth films.

Samples were spiked with 10 µg/mL of Cd (II) and 10 µg/mL of Pb (II) when paper electrodes were modified with mercury films and with 25 µg/mL of each analyte for bismuth films.

For the determination on paper electrodes modified with mercury films, standard additions were made with solutions prepared with an initial aliquot of 100 µL of the spiked sample (10 µg/mL of Cd (II) and 10 µg/mL of Pb (II)). Then, different aliquots (100, 200, 300 and 400 µL) of a 10 µg/mL of Cd (II) and Pb (II) standard solution in 0.1 M acetate buffer (pH 4) with 0.5 M sodium sulphate as a background electrolyte were added. Finally, buffer solution was added until a final volume of 1 mL. Solutions were analyzed by the stripping voltammetry method developed. The quantification of both analytes was successfully carried out at mercury films. However, Pb (II) and Cd (II) quantification on paper electrodes modified with bismuth films was quite irreproducible. This could be due to the formation of intermetallic compounds (Pb/Cd) between the oxidation peaks for Pb (II) and Cd (II). This could alter the intensity of the peaks analyzed yielding lower concentration in solution for both heavy metals. It has been described that the presence of Zn (II) in the solution could avoid the formation of those intermetallic compounds with Cd and Pb and eliminate this interference [36]. Then, for the determination of Cd (II) and Pb (II) on paper-based electrodes modified with bismuth films, Zn (II) was added to the solutions employed for the determination, following the standard addition methodology. Therefore, the determination procedure was modified according to the method previously published [36].

With this aim, samples of water were spiked with Zn (II), Cd (II) and Pb (II) standards, obtaining a spiked sample of 25 µg/mL of Zn (II), 25 µg/mL Cd (II) and 25 µg/mL Pb (II) in 0.1 M acetate buffer pH 4 with 0.5 M sodium sulphate. For the determination on paper electrodes modified with bismuth films, standard additions were carried out by taking 100 µL of the spiked sample. Then, different aliquots (100, 200, 300 and 400 µL) of a standard solution with 10 µg/mL of Cd (II) and 10 µg/mL of Pb (II) were added. Finally, buffer solution was added until a final volume of 1 mL. Solutions were analyzed by stripping voltammetry with the next parameters: preconcentration step at −1.3 V for 600 s, followed by anodic stripping linear sweep voltammetry and scanning the potential from −1.3 to 0 V at 50 mV/s with 2 mV of step potential. With this procedure, samples of water spiked with Cd (II) and Pb (II) were analyzed with a reproducibility similar to that obtained for mercury films.

Results obtained indicated a higher recovery of the spiked sample in analysis made on paper electrodes modified with mercury films in comparison with bismuth films. Pb (II) determination had a recovery of 98.8% on mercury films, whereas a recovery of 89.2% was obtained on bismuth films. Recoveries obtained for Cd (II) determination were of 99.2% and 86.3%, concluding the efficacy of using paper electrodes with mercury films. The results obtained for the sample with paper-based electrodes with mercury films developed in this study are similar to those obtained on an analysis of the sample by a method previously developed at SPCEs modified with bismuth films (103% Pb (II), 97.5% Cd (II)) [71]. In this case, the possibility of reusing the SPCEs with paper electrodes allows the development of a sensor with a lower cost.

### 3.4. Comparison of Methods

The analytical characteristics reported in the literature for trace metals’ determination by using paper-based electrodes are shown in Table 2. Heavy metal determination on paper devices was carried out by different physicochemical principles, mainly spectrophotometric and electrochemical. However, electrochemical devices can be used for the quantification of metals with less reagent consumption: only those for the formation of the metallic films and electrolytes; therefore, it could be considered that electrochemical methods offer a more economic option. In addition, this methodology was tested with a higher variety of analytes obtaining characteristics like linear range, sensitivity and reproducibility, in comparison with devices based on a colorimetric determination (Table 2).

Electrochemical devices for heavy metal determination in paper devices are scarce. The LOD of our procedure might seem high compared with some electrochemical paper devices previously published, but with the method developed in this work, other analytes, such as Cu (II) and In (III), could be successfully analyzed, increasing the scope of application for this heavy metal sensor. The LOD for Pb (II) and Cd (II) make it suitable for monitoring the levels in contaminated water. In the case of Cu (II), the linear range obtained, together with the high reproducibility, is a promising low-cost alternative to colorimetric sensors. Our work has an additional advantage regarding waste management, since all heavy metals analyzed, as well as the films formed, stay in the working paper electrode. Therefore, the SPCE platform can be reused without any contamination, and the paper electrodes used can be easily disposed of due to their small size and easy portability.

## 4. Conclusions

An analytical methodology for the study of heavy metals in paper electrodes modified with mercury or bismuth films was successfully developed. Films were freshly generated by applying a reduction potential in solutions containing mercury (II) or bismuth (III) ions, in order to develop a paper platform to quantify heavy metals. The paper working electrodes were overlapped on the working electrode of a screen-printed card. This working-to-working configuration provided the following: (i) a homogeneous connection for all the electrode’s circular area; (ii) the possibility of reusing the screen-printed card, since the ink in the paper covers the pores and the ink in the ceramic card is not wetted; and (iii) auxiliary and reference electrodes required for film generation and further measurements. Cd (II), Pb (II) and In (III) could be detected in both films, whereas Cu (II) could only be detected in paper electrodes modified with mercury films. Furthermore, mercury films offered higher sensitivity for all heavy metals tested, in comparison with paper electrodes modified with bismuth films, with limits of detection of 0.4, 0.1, 0.04 and 0.2 µg/mL for Cd (II), Pb (II), In (III) and Cu (II). Cd (II) and Pb (II) could be determined in spiked water samples with recoveries of 99.2% and 98.8% for mercury films and 88.7% and 89.2% for bismuth films. Both types of paper-based electrodes modified with films could be considered as low-cost sensors to the determination of heavy metals in contaminated water. Analytical characteristics like linear range, sensitivity and reproducibility were calculated in a higher variety of metals than paper sensors based on colorimetric detection. Mercury films were a better option to determine heavy metals, and bismuth films were a more sustainable option. The small size of the platform makes it very manageable and easy to treat in terms of waste management.

## Figures and Tables

**Table 1:** Analytical characteristics for Cd (II), Pb (II), In (III) and Cu (II) on paper-based electrodes modified with bismuth or mercury films.

**Bismuth Films**			
	**RSD (%)**	**Linear Range (µg/mL)**	**Limit of Detection (µg/mL)**
Cd	4.3	2.5–10	1
Pb	14.6	1–10	0.7
In	9.6	1–4	0.6
**Mercury films**			
	RSD (%)	Lineal range (µg/mL)	Limit of detection (µg/mL)
Cd	6.4	0.5–10	0.4
Pb	10.9	0.5–10	0.1
In	9.1	0.1–5	0.04
Cu	4.3	0.25–6.35	0.2

**Table 2:** Analytical characteristics of other paper devices described in the literature.

Sensor	Detection Technique	Analyte	Linear Range (µg/mL)	LOD (µg/mL)
Solid phase extraction-µPADs	Colorimetric [72]	Cu (II)	0.02–500	0.02
Rotational paper-based device	Colorimetric [73]	Ni (II)	1.5–60	4.8
		Cu (II)	0.5–80	1.6
		Cr (VI)	0.5–10	0.18
Table style paper device	Colorimetric [66]	Ni (II)	0.3–5	0.3
		Cu (II)	0.6–3	0.6
		Cr (VI)	0.2–3	0.2
Three-dimensional microfluidic device	Colorimetric [58]	Cu (II)	5–20	0.29
		Cd (II)	0.05–0.4	0.19
		Ni (II)	5–20	0.33
		Cr (VI)	0.2–1	0.35
Double-sided conductive adhesive carbon tape with bismuth	Electrochemical (SWASV) [62]	Pb (II)	0.002–0.5	0.002
		Cd (II)	0.1–0.2	0.1
		Zn (II)	0.1–0.2	0.1
BDDPE-µPADs	Electrochemical (SWASV) [61]	Pb (II)	0.001–0.2	0.001
		Cd (II)	0.025–0.2	0.025
Graphite paper electrode with sulfonated polyaniline/antimony	Electrochemical (DPASV) [74]	Pb (II)	0.002–0.07	0.0002
		Cd (II)	0.002–0.07	0.00041
Electrochemical device with silver ink and office paper	Electrochemical (SWASV) [75]	Pb (II)	1.87–9.95	0.35
